# Non-Molecular-Clock-Like Evolution following Viral Origins in *Homo sapiens*

**Published:** 2007-09-26

**Authors:** Wendy Mok, Kelly Seto, Jon Stone

**Affiliations:** 1 Department of Biochemistry and Biomedical Sciences, McMaster University, 1280 Main Street West, Hamilton ON L8S 4K1, Canada.; 2 Department of Molecular and Medical Genetics, University of Toronto, 1 King’s College Circle, Toronto ON M5S 1A8, Canada.; 3 Department of Biology and Origins Institute, McMaster University, 1280 Main Street West, Hamilton ON L8S 4K1, Canada.

**Keywords:** computational biology, epidemic, mutation, virus, SARS-CoV

## Abstract

Researchers routinely adopt molecular clock assumptions in conducting sequence analyses to estimate dates for viral origins in humans. We used computational methods to examine the extent to which this practice can result in inaccurate ‘retrodiction.’ Failing to account for dynamic molecular evolution can affect greatly estimating index case dates, resulting in an overestimated age for the SARS-CoV-human infection, for instance.

## Introduction

Dating when viruses acquired the ability to infect human genomes is paramount to managing public health. Infections could occur directly and repeatedly via other animal hosts (e.g. Human Immunodeficiency Virus, West Nile Virus). But, if infections were manifested secondarily, as a consequence from viral sequence substitutions that allowed sustained transmission among humans—as recent reports suggest for Avian Influenza Virus—a pandemic could ensue, with casualty numbers greatly exceeding those for influenza pandemics from the past century (Nicholls, 2006; Thomas and Noppenberger, 2007).

Pinpointing viral origins in humans enables researchers to extrapolate backward to estimate index case dates, calculate mutation and substitution rates, and document genetic events that permit efficient interspecies transmission and enhanced virulence (Chen et al. 2004). These data enable researchers to extrapolate forward to predict variability and develop vaccination or management programs to prevent or respond to potential global outbreaks. Herein, we show that adopting molecular clock assumptions can yield inaccurate estimated origin times, considering as an example data from the Severe Acute Respiratory Syndrome coronavirus (SARS-CoV) infection in humans.

**Figure 1.**
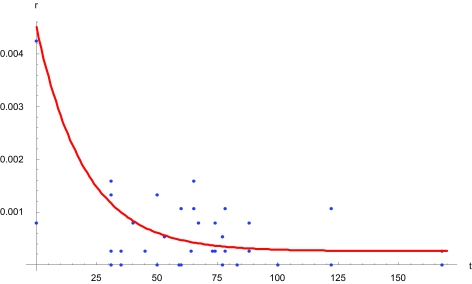
Quantifying Severe Acute Respiratory Syndrome coronavirus (SARS-CoV) sequence modification over time. The points represent nucleotide substitution rates r among 51 SARS-CoV sequences obtained between November 2002 and March 2003, inferred on the basis of a recently published phylogenetic tree (He et al. 2004). The upper and lower points at t = 0 represent r (civet sequence SZ16, human sequence GZ02) and r (human sequence GZ02, human sequence GD01), respectively. The curve is a gamma distribution function that is similar to the gamma distribution function that was used in computer simulations. t = time (days since estimated initial transmission from civets to humans); r = sequence divergence rate (substitutions per site per evolutionary step).

We compared the S-gene in SARS-CoV sequences isolated from patients included in a recently published phylogenetic tree (He et al. 2004). This gene synthesizes the Spike-protein that is involved in virus-to-host-cell-epitope interactions, so sequence changes will affect evolutionary dynamics. We observed that stepwise genetic distance was greatest immediately following initial infection and diminished to a plateau then after (Fig. 1). Recognizing that this change in substitution rate would violate a molecular clock assumption and could cause pairwise genetic distances to yield inaccurate evolutionary divergence estimates (especially if genetic distance calculations were performed with respect to a reference sequence representing an hypothetical common ancestor), we quantified the extent to which failing to account for dynamic SARS-CoV evolution might affect estimating an origin time.

**Figure 2.**
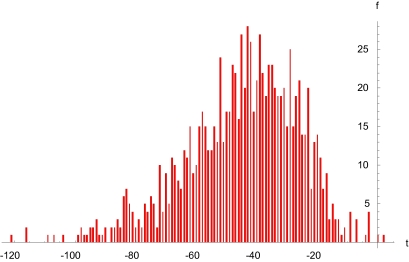
Estimated origin times for Severe Acute Respiratory Syndrome coronavirus (SARS-CoV) in humans. The distribution was obtained using a computer simulation program that evolved virtually on the basis of a recently published phylogenetic tree (He et al. 2004) 51 SARS-CoV sequences (1000 replicates); performed a linear regression involving divergence times and genetic distances from a hypothetical ancestor; and extrapolated backward to 0 divergence to obtain estimated origin times t.

We developed a computer simulation program to emulate sequence evolution and used it to evolve *in silico* and according to the recently published phylogenetic tree (He et al. 2004) the SARS-CoV sequences. The computer simulation program determined substitution rates on the basis of a gamma distribution function (Fig. 1). It implemented prescriptions for designating time-points for events, such as strains becoming ‘extinct’ in patients, to accord as closely as possible to definite time-points, such as dates on which patients died. And it determined origin times according to a molecular clock assumption. We ran 1000 replicates to obtain an origin time distribution, from which we could obtain a representative, estimated (e.g. median) index case date (Fig. 2).

## Materials and Methods

We obtained from Genbank 51 SARS-CoV sequences isolated from infected patients at different times throughout the epidemic and included in the aforementioned published phylogenetic tree (He et al. 2004). These constituted the available, unique sequences from the 61 that were included in that phylogenetic tree. We extracted from each among the 51 patient sequences a 3767-nucleotide sequence encoding the Spike (S) protein. We aligned these sequences using ClustalX (Thompson et al. 1997) and determined stepwise genetic distances between sequentially emerging strains (i.e. according to the phylogenetic tree) to calculate substitution rates (nucleotides per site per day).

We used the observed substitution rate variation (Fig. 1) and independently published average mutation rates (He et al. 2004; Lu et al. 2004) to define a gamma distribution function (e.g. Golding, 1983) relating time (days) to expected genetic distance (substitutions per site). We used this gamma function (α = 1, θ = 4, implemented using the function Gamma Distribution[1, 4] + 0.18 in *Mathematica* (Wolfram Research Inc, 1988)) in the computer simulation program to evolve sequences according to the phylogenetic tree, which was rooted by using as outgroups sequences obtained from strains found in civet cats (He et al. 2004). Comparable results were obtained using one- and two-parameter molecular substitution models (Jukes and Cantor, 1969; Kimura, 1980). Reassortment was unconsidered. The computer simulation program calculated Hamming distances between an hypothetical ancestor and the sequences; paired time points with these distances according to the phylogenetic tree; performed linear regressions; and extrapolated backward to 0 distance, to estimate origin times. We ran 1000 replicates and determined the median and 95% confidence interval for the resulting distribution (Fig. 2), which allowed us to estimate a representative index case date.

## Results

In our computer simulations, the origin time for SARS-CoV in humans was estimated to have transpired approximately 45 days (median t = −45, Fig. 2) prior to the actual index case date (95% confidence interval: −85, −15 days). This would correspond to mid September-mid October, 2002; December 31, 2002; or January 26, 2003 for previously published real-world estimates (August-September 2002 (Lu et al. 2003); November 16, 2002 (He et al. 2004); and December 12, 2002 (Zeng et al. 2003)).

## Discussion

Adopting a molecular clock assumption generated inaccurate estimated origin times for virtual SARS-CoV infections in humans, yielding estimated initial infection dates that differed in comparison to previously published estimates for actual index case dates. We note that previously published estimates ‘redate’ inaccurately, to months prior to or weeks following the real-world index case date (He et al. 2004; Lu et al. 2004) or months following the initial outbreak (Zeng et al. 2003). Those estimates were generated using sequences that were obtained between February and April 2003, whereupon molecular modification had stabilized (Fig. 1). Adopting a molecular clock assumption might have been valid in those proficient analyses; however, extrapolating backward on the basis of that constant modification rate—especially using pairwise genetic distances—might have been misleading.

We propose that the nonlinear, rapid divergence exhibited by the SARS-CoV immediately after initially infecting humans (t = 0–25, Fig. 1) might represent a time period during which the virus ‘settled’ before becoming ‘comfortable’ (t >25, Fig. 1) in its new environment. Whether settling constitutes a general phenomenon could be tested with data from entire genomes (i.e. including all genes) and other viruses (e.g. the Avian Influenza Virus). In the meanwhile, we recommend subjecting virus sequences to computational, non-molecular clock assumption analyses (e.g. “relaxed phylogenetics” in Drummond et al. 2006) to estimate time-points for critical epidemiological phenomena, like the viral reassortment events associated with the first human SARS-CoV case in 1997 (He et al. 2004).
